# A class C CpG toll-like receptor 9 agonist successfully induces robust interferon-alpha production by plasmacytoid dendritic cells from patients chronically infected with hepatitis C

**DOI:** 10.1111/j.1365-2893.2008.01011.x

**Published:** 2009-05

**Authors:** N A Libri, S J Barker, W M C Rosenberg, A E Semper

**Affiliations:** 1 iQur Ltd, Southampton General HospitalSouthampton, UK; 2 Liver Group, Division of Infection Inflammation and Repair, University of Southampton, Southampton General HospitalSouthampton, UK

**Keywords:** CpG, dendritic cell, hepatitis C, interferon-α, plasmacytoid, toll-like receptor

## Abstract

Immunomodulators that induce local endogenous interferon-alpha (IFN-α) production by plasmacytoid dendritic cells (pDCs) may offer new strategies for the treatment of patients chronically infected with the hepatitis C virus (HCV). However, such an approach may be compromised if reports are true that IFN-α production by pDCs from patients with chronic HCV (cHCV) is profoundly impaired. To address the question of pDC dysfunction in cHCV more definitively, in the present study a panel of four prototypic synthetic agonists of toll-like receptor 7 (TLR7) or TLR9 were administered *in vitro* to pDCs purified from cHCV patients and from normal uninfected donors and their responses compared in terms of not only IFN-α production but also the global expression of other cytokines and phenotypic maturation. Plasmacytoid DCs from uninfected donors produced substantial levels of IFN-α in response to three of the four agonists and yet only one TLR9 agonist, a class C CpG oligodeoxynucleotide (ODN), induced robust IFN-α production by pDCs from cHCV patients. Proinflammatory cytokine production and phenotypic maturation in response to all four agonists was equivalent in infected and uninfected pDCs. These data point to a profound but selective defect in IFN-α production by pDCs from cHCV donors. Nonetheless, a class C CpG ODN successfully induced robust IFN-α production, suggesting that this class of TLR9 agonist may have utility as a future immunotherapeutic for the treatment of chronic HCV infection.

## Introduction

The hepatitis C virus (HCV) is a blood-borne, hepatotrophic virus which establishes chronic HCV (cHCV) infection in up to 85% of cases. With an estimated 2% of the global population infected with HCV [1], the potential for chronic infection to lead to cirrhosis, end-stage liver disease and hepatocellular carcinoma, poses a considerable health burden.

The mainstay of current therapies for cHCV is interferon alpha (IFN-α), a type 1 interferon typically administered as pegylated, single-species IFN-α2 in combination with the antiviral ribavirin. IFN-α has multiple antiviral actions [2]. It induces genes that inhibit viral replication [3] and boosts the innate and adaptive immune response, activating NK cells [4] and myeloid DCs [5] respectively. IFN-α also promotes the development of a polarized antigen-specific type 1 T-cell response [6,7] thought to favour resolution of HCV infection [8] and pDC-derived IFN-α supports the induction of CD8^+^ T cell responses [9]. Nonetheless, combined pegylated IFN-α2/ribavirin therapy succeeds in only 42–46% of HCV genotype 1 infections and 76–80% of genotype 2/3 infections [2]. Furthermore, the raised systemic levels of IFN-α can lead to psychiatric and other adverse events necessitating premature cessation of treatment in approximately 10% of cases [10]. Alternative novel strategies for the treatment of cHCV are therefore required.

One approach being explored is the use of immunomodulators to induce endogenous, local IFN-α production by plasmacytoid dendritic cells (pDCs). In response to viral infection, pDCs are stimulated to migrate to lymph nodes where they produce large amounts of type I interferon (IFN-α, IFN-β, IFN-ω and IFN-λ) and a broad spectrum of IFN-α subtypes [11–13]. Plasmacytoid DCs sense viral infection through innate pattern recognition receptors of the toll-like receptor (TLR) family, specifically TLR7 and TLR9 [14]. Upon ligation, TLR7 and TLR9 trigger a cascade of shared intracellular signalling events which culminate in abundant IFN-α production together with enhanced production of other cytokines and pDC maturation [15,16]. Thus, TLR7 or TLR9 may be appropriate pharmacological targets for immunomodulation of IFN-α production by pDCs, with the diverse range of endogenous type I IFN species produced offering an advantage over the administration of an exogenous single species of IFN-α.

The natural ligands for TLR7 are patterns within viral single-stranded RNAs [17] but synthetic ligands including imidazoquinolines [18] and guanosine analogues [19] are also recognized. TLR9 recognizes unmethylated CpG dinucleotide-rich sequences within bacterial and viral DNA, which can be mimicked by synthetic CpG oligodeoxynucleotides (ODNs) [20]. Three categories of CpG-ODNs have been identified: classes A (or ‘D’), B (or ‘K’) and C. CpG-A ODNs favour IFN-α production over pDC differentiation and are poor B-cell activators, whereas CpG-B ODNs show the converse [21]. CpG-C ODNs combine strong IFN-α induction and efficient pDC maturation [22,23] and efficiently stimulate B cells.

A possible hurdle to using TLR7 or TLR9 agonists as inducers of endogenous IFN-α production in cHCV are reports that pDCs derived from cHCV patients are profoundly impaired in their ability to produce IFN-α upon *in vitro* stimulation [24–31]. However, other studies have found IFN-α production by cHCV–pDCs to be intact [32–35]. Therefore, the purpose of the current study was to use a panel of prototypic synthetic TLR7 and TLR9 agonists to determine if normal function can successfully be induced in pDCs taken from cHCV subjects. The TLR7 agonists selected were loxoribine (a guanosine analogue) and imiquimod (an imidazoquinoline). The TLR9 agonists were the class A CpG, ODN2216 [36] and the class C CpG, M362 [22]. The function of pDCs purified from chronically-HCV infected and normal, uninfected subjects was investigated not only in terms of IFN-α production, but also with respect to the production of a range of other cytokines and pDC maturation.

## Materials and Methods

### Study subjects

Ethical approval was obtained from Southampton and South West Hampshire Joint Research Ethics Committee. Thirty-seven patients with detectable HCV RNA and a clinical diagnosis of cHCV disease were recruited from hepatology clinics run by Southampton University Hospitals NHS Trust. All gave written informed consent. Patients treated for HCV within the preceding 6 months or known to be coinfected with another blood-borne virus were excluded from the study. Table 1 summarizes the clinical characteristics of the cHCV patients and the 25 normal healthy donors (NHD) used as controls. NHD subjects had no known risk factors for blood-borne virus infection.

**Table 1:** Subject clinical characteristics

Characteristics	cHCV patients (*n*=37)	Normal healthy subjects (*n*=25)
Age (years); mean (range)	49 (36–75)	48 (23–75)
Gender (M/F)	27/10	20/5
ALT (IU/L); mean (range)	88 (18–278)	N/A
HCV viral load (IU/mL); mean (range)*	4.86 × 10^6^ (1.02 × 10^4^−3.01 × 10^7^)	N/A
Genotype†		
1	20	N/A
2/3	13	
4	1	
Non-1	3	
Disease severity‡		
Mild	16	N/A
Mild/moderate	4	
Moderate	10	
Severe (+cirrhosis)	7 (6)	
Treatment naïve/non-responder	24/13	N/A

* Viral load determined for 31 patients by quantitative PCR (Cobas Amplicor HCV Monitor test; Roche Molecular Systems, Pleasanton, CA, USA). In the remaining six patients, presence of HCV virus was confirmed using a qualitative PCR assay.

† HCV genotype determined using PCR-based ‘Genotyping for Treatment Assay’ (iQur Ltd, Southampton, UK), with exception of ‘non-1’ samples which used a superceded assay.

‡ Based on histological analysis of biopsies for inflammation and fibrosis. In the absence of histological evidence, disease status was determined by clinical criteria including physical examination, diagnostic imaging and laboratory indices.

### Plasmacytoid DC preparation

Peripheral blood mononuclear cells (PBMCs) were separated from fresh EDTA-anticoagulated blood obtained from cHCV patients (typically 25–50 mL) or uninfected subjects (100–400 mL), by Lymphoprep (Robbins Scientific, Solihull, UK) density gradient centrifugation. Plasmacytoid DCs (89–95% pure) were isolated from PBMCs by positive immunomagnetic selection with anti-CD304 (BDCA-4)-coated immunomagnetic beads (Miltenyi Biotec, Bisley, UK).

### Stimulation of plasmacytoid DCs

Freshly-isolated pDCs (2 × 10^5^/mL) in complete RPMI medium [RPMI 1640 without phenol red with 2 mm l-glutamine, 100 U/mL penicillin, 100 μg/mL streptomycin (all Invitrogen Ltd, Paisley, UK) and 10% heat-inactivated FCS (Hyclone, Perbio Science UK Ltd, Tattenhall, UK)] were plated into 96-well (200 μL/well) or 48-well (500 μL/well) plates, depending on cell yields. pDCs were cultured alone or with CpG-A (ODN 2216, [36]), ODN 2216 control (GpC control for ODN2216), CpG-C (M362, [22]), loxoribine or imiquimod at the concentrations indicated in the text. All TLR agonists were from InvivoGen (San Diego, CA, USA) and were reconstituted in endotoxin-free water. After 20–24 h incubation at 37 °C in 5% CO_2_, cell-free supernatants were collected and frozen pending cytokine analysis and/or pDCs were retained for phenotypic analysis.

### Quantifying cytokine production

Secreted IFN-α was measured using a human IFN-α multi-species ELISA (PBL Biomedical Laboratories, Piscataway, NJ, USA) which recognizes 13 of 14 IFN-α isoforms, the exception being IFN-αF. The manufacturer’s ‘extended range’ protocol was followed.

Human IFN-γ, IL-1β, IL-2, IL-4, IL-5, IL-6, IL-8, IL-10, IL-12p70 and TNF-α were measured in pDC culture supernatants using a multiplex fluorescent bead immunoassay (Bender MedSystems, Vienna, Austria). Supernatants were incubated in 96-well filter plates with a cocktail of analyte-specific beads and biotinylated secondary antibodies, according to the manufacturer’s standard procedures, followed by detection with streptavidin-PE. The mean fluorescence intensity (MFI) of 3000 bead events was measured using a FACSCalibur flow cytometer with CellQuest software (BD Biosciences, Oxford, UK). Analyte concentration was determined by reference to a standard curve.

### Phenotypic analysis of plasmacytoid DCs

Cells in PBS containing 0.1% NaN_3_, 1% BSA and 100 μg/mL human IgG (Jackson Immunoresearch, West Grove, PA, USA) were blocked for 15 min on ice then labelled with monoclonal antibodies against HLA-DR FITC (clone L243), CD86-FITC (clone FUN-1), CD83-APC (clone HB15e) (all from BD Biosciences) or CD303-APC (BDCA2, clone AC144; Miltenyi Biotec) or with isotype controls [murine IgG1 (clone MOPC 31, APC or FITC conjugated) or IgG2a FITC (clone G155-178) (all from BD Biosciences)]. After staining, pDCs were washed, fixed in 1% formaldehyde and stored on ice in the dark pending data acquisition using a FACSCalibur flow cytometer. MFI values for cell surface marker staining were first corrected by subtracting the MFI values of the relevant isotype controls. Data are expressed as the fold change in corrected MFI between unstimulated and agonist-stimulated cells.

### Statistical analysis

Statistical analyses were performed using GraphPad Prism version 4.0 for Windows (GraphPad Software, San Diego, CA, USA). Having verified that the data were normally distributed, statistically significant differences between the mean responses of NHD and cHCV subjects and between the mean responses to agonist stimulation, were determined using two-way anova, with Bonferroni post-tests.

## Results

### Depressed IFN-α production by pDCs from patients with chronic HCV-infection

**Fig. 1:**
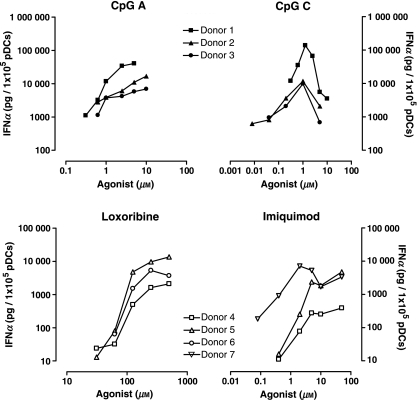
IFN-α production by NHD-pDCs in response to TLR7 and TLR9 agonist dose. Purified pDCs from three uninfected donors were stimulated for 24 h with a dose range of CpG-A or CpG-C. NHD-pDCs from a further four donors were stimulated with loxoribine or imiquimod. Secreted IFN-α was measured using a multi-subtype ELISA. The optimal dose of each agonist was determined and is stated in the text.

Plasmacytoid DCs circulate in the blood at low frequency and pDC numbers may be further reduced in cHCV infection [25,27]. With low volumes of cHCV blood (≥25 mL), pDC numbers would be insufficient to use each TLR agonist at a range of doses. Therefore, the optimal dose for IFN-α induction by each agonist was first determined using NHD–pDCs. For CpG-C, 1 μm was selected as the optimal dose since it induced maximal levels of IFN-α which declined sharply at higher concentrations of CpG-C (Fig. 1). By contrast, IFN-α levels declined steadily with decreasing concentrations of CpG-A, falling off more markedly below 1 μm (Fig. 1). Therefore, 1 μm was also selected as the optimal working concentration for CpG-A, in line with published studies in both NHD- [21,37] and cHCV-pDCs [25,29,35]. Based on their stimulation of IFN-α release by NHD-pDCs, 250 μm loxoribine and 5 μm imiquimod were selected as optimal working concentrations (Fig. 1).

**Fig. 2:**
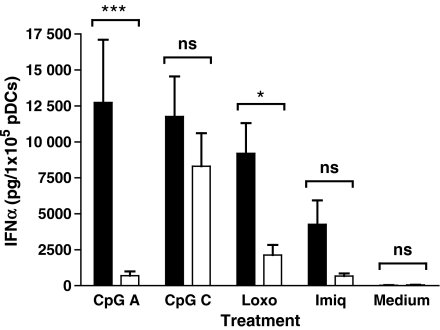
Deficit in TLR7 and TLR9-induced IFN-α production by pDCs in chronic HCV infection. Purified pDCs from uninfected (NHD, solid bars) and HCV-infected (cHCV, open bars) donors were stimulated for 20–24 h with medium alone (NHD, *n*=19; cHCV, *n*=25) or with 1 μm CpG-A ODN (NHD, *n*=13; cHCV, *n*=20), 1 μm CpG-C ODN (NHD, *n*=15; cHCV, *n*=20), 250 μm loxoribine (NHD, *n*=17; cHCV, *n*=20) or 5 μm imiquimod (NHD, *n*=16; cHCV, *n*=17) and secreted IFN-α assayed using a multi-subtype ELISA. Bars represent means ± SEM. Compared to culture in the absence of a TLR agonist (medium), NHD–pDCs produced significant quantities of IFN-α in response to CpG-A (*P*<0.001), CpG-C (*P*<0.001) and loxoribine (*P*<0.001). IFN-α production upon stimulation with the TLR9 agonists was significantly higher than following imiquimod treatment (CpG-A > imiquimod, *P*<0.01; CpG-C > imiquimod, *P*<0.01). Robust IFN-α production by cHCV–pDCs only occurred in response to CpG-C, reaching levels significantly higher than any other treatment (CpG-C > medium, *P*<0.001; CpG-C > CpG-A, *P*<0.01; CpG-C > loxoribine, *P*<0.05; CpG-C > imiquimod, *P*<0.01). Significant differences in the response to individual agonists are shown on the graph: CpG-A, cHCV-pDCs < NHD-pDCs, ***P<0.001; loxoribine, cHCV–pDCs < NHD–pDCs, *P<0.05. The response of NHD– and cHCV–pDCs to CpG-C did not differ significantly (n.s., *P*>0.05) nor to imiquimod (*P*>0.05) or medium (*P*>0.05).

Purified pDCs from cHCV patients and uninfected volunteers were cultured for 20–24 h alone or with 1 μm CpG-A, 1 μm CpG-C, 250 μm loxoribine or 5 μm imiquimod. In preliminary experiments, an equimolar amount of control CpG-A ODN in which all CpG dinucleotides were reversed was included. NHD–pDCs released negligible levels of IFN-α when cultured alone (30.51 ± 7.00 pg/10^5^ cells, Fig. 2) or with the control ODN (11.90 ± 4.60 pg/10^5^ cells, data not shown). Compared to the medium-only control, CpG-A, CpG-C and loxoribine induced significant levels of IFN-α (Fig. 2 and legend) and were of similar potency, whereas imiquimod stimulated only weak IFN-α production to levels that were not statistically significant.

In striking contrast, in HCV infection, statistically significant IFN-α production by cHCV-pDCs was only induced by CpG-C (Fig. 2), reaching levels equivalent to those induced in uninfected pDCs. Compared to NHD-pDCs, IFN-α production by cHCV-pDCs was significantly depressed in response to CpG-A and loxoribine (Fig. 2) with mean levels of IFN-α production by cHCV-pDCs 94.6% lower in response to CpG-A and 76.9% lower in response to loxoribine. In response to imiquimod, although cHCV–pDCs produced mean levels of IFN-α 84.2% below normal (NHD, 4266 ± 1665; cHCV, 675.6 ± 180.1), the difference between the two subject groups was not statistically significant (*P*>0.05).

Dividing the cohort of cHCV patients into those who had failed to respond to previous treatment with IFN-α2/ribavirin (non-responders, NR) and patients who had not yet received treatment (treatment naïve), no significant difference was found between the two groups in terms of mean IFN-α production in response to CpG-A, CpG-C or loxoribine [CpG-A: NR (*n* = 7), 364.3 ± 121.4 *vs* naïve (*n* = 13), 877.8 ± 453.6, *P* = 0.4271; CpG-C: NR (*n*=7), 10720 ± 3455 *vs* naïve (*n*=13), 7015 ± 3033, *P*=0.4558; loxoribine: NR (*n*=9), 1537 ± 613.0 *vs* naïve (*n*=11), 2606 ± 1199, *P*=0.4680]. Thus, both patient subsets failed to respond to CpG-A and loxoribine, but mounted robust IFN-α production following stimulation with CpG-C.

### Cytokine production by pDCs from patients with chronic HCV-infection is not globally impaired

**Fig. 3:**
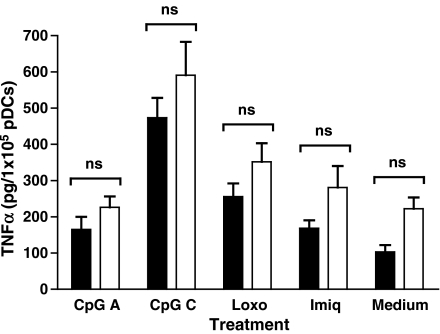
TLR7 and TLR9 agonists induce normal levels of TNF-α production in pDCs from chronically HCV-infected subjects. pDC supernatants from a subset of the NHD and cHCV subjects shown in Fig. 2 were additionally assayed for the presence of TNF-α, yielding the following data sets: medium alone (NHD, *n*=10; cHCV, *n*=15), 1 μm CpG-A ODN (NHD, n = 9; cHCV, *n*=9), 1 μm CpG-C ODN (NHD, *n*=9; cHCV, *n*=10), 250 μm loxoribine (NHD, *n*=10; cHCV, *n*=11) and 5 μm imiquimod (NHD, *n*=10; cHCV, *n*=9). Bars represent means ± SEM; NHD (solid bars), CHC (open bars). Compared to cells exposed to medium alone, TNF-α was significantly induced only by CpG-C (*P*<0.001). In both NHD- and cHCV-pDCs, CpG-C induced significantly more TNF-α than CpG-A (*P*<0.001), loxoribine (*P*<0.01) or imiquimod (*P*<0.001). The response of NHD- and cHCV–pDCs to individual agonists did not differ significantly (n.s.).

Although IFN-α is the principle cytokine produced by pDCs, they can also produce TNF-α, IL-6 and IL-8 and in some cases, IL-12 and IL-10. Using a multiplex assay, these cytokines were analysed in the same supernatants used for IFN-α measurements. In both NHD- and cHCV–pDCs, only TNF-α was significantly induced above control levels and only in response to the CpG-C ODN (Fig. 3). Comparing TNF-α production by pDCs from the two subject groups for individual TLR agonists, the mean levels of TNF-α production did not differ significantly (Fig. 3) although there was a consistent trend for TNF-α to be expressed at higher levels by cHCV- than NHD-pDCs (Fig. 3).

In both NHD- and cHCV-pDCs, release of IL-6, IL-8 and IL-10 was enhanced by some TLR agonists, but these increases were not statistically significant (Table 2). In terms of IL-6 and IL-10 production, cHCV- and NHD–pDCs functioned equally but basal and induced levels of IL-8 expression were consistently, although non-significantly, higher in cHCV- than NHD-pDCs (Table 2). IL-12 was not induced by any of the agonists (data not shown).

**Table 2:** Expression of IL-6, IL-8 and IL-10 in response to TLR agonists

	IL-6			IL-8			IL-10		
Stimulus	NHD	cHCV	P*	NHD	cHCV	P*	NHD	cHCV	P*
CpG-A	3268 ± 1399	2142 ± 664.6	n.s.	7263 ± 1437	12080 ± 2480	n.s.	365.9 ± 212.3	356.8 ± 136.3	n.s.
CpG-C	4429 ± 1467	3817 ± 1074	n.s.	9034 ± 1876	12320 ± 1631	n.s.	385.7 ± 169.7	397.7 ± 176.3	n.s.
LOXO	2545 ± 1060	1739 ± 686.1	n.s.	10380 ± 2931	14750 ± 3385	n.s.	224.6 ± 94.48	274.1 ± 137.7	n.s.
IMIQ	2292 ± 797.7	2404 ± 1320	n.s.	8354 ± 1587	15250 ± 5062	n.s.	216.7 ± 83.90	349.1 ± 147.0	n.s.
Medium	1774 ± 738.2	1555 ± 700.9	n.s.	7703 ± 1662	11410 ± 3138	n.s.	126.4 ± 47.89	144.7 ± 39.77	n.s.

Values are expressed as mean ± SEM.

* Significance of difference between mean cytokine production by NHD and cHCV–pDCs for each TLR agonist, determined by two-way anova with Bonferroni post-test.

### *In vitro* immunophenotypic maturation of pDCs is not impaired in cHCV infection

Upon stimulation, as well as producing type I IFN, pDCs differentiate into mature DCs capable of antigen presentation [38]. To determine if pDCs from cHCV patients undergo normal immunophenotypic maturation in response to TLR7 and TLR9 agonists, changes in expression of CD86, HLA-DR, CD83 and BDCA-2 were measured.

**Fig. 4:**
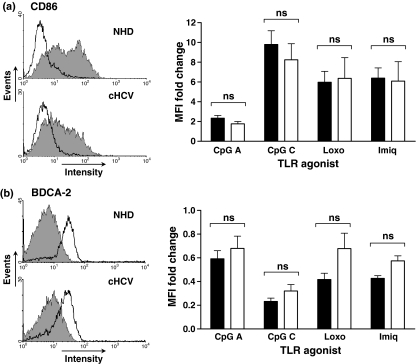
Immunophenotypic maturation of pDCs is not impaired in cHCV infection. After 20–24 h stimulation with TLR agonists, pDCs were processed for flow cytometry to identify immunophenotypic changes consistent with maturation. FACS histograms from a representative NHD and cHCV subject show (a) the rise in CD86 expression and (b) fall in BDCA-2 expression following stimulation with CpG-C (solid histogram) compared to unstimulated cells (open histogram overlay). The bar graphs show the average fold change in mean fluorescence intensity (MFI) ± SEM of (a) CD86 and (b) BDCA-2 on pDCs from 9 NHD (solid bars) and eight cHCV (open bars) subjects (seven NHD for TLR7 agonists) induced by the four TLR agonists. For each TLR agonist, the degree of (a) upregulation of CD86 and (b) downregulation of BDCA-2 did not differ significantly between NHD–pDC and cHCV–pDCs (n.s.). CpG-C was a significantly stronger stimulus for CD86 upregulation than CpG-A (NHD–pDCs, *P*<0.001; cHCV–pDCs, *P*<0.01) and for cHCV–pDCs loxoribine was also significantly more potent than CpG-A (*P*<0.05). CpG-C caused significantly stronger downregulation of BDCA-2 than CpG-A (NHD–pDCs, *P*<0.01; cHVC–pDCs, *P*<0.01).

After 20–24 h of stimulation, phenotypic maturation was equivalent for NHD- and cHCV–pDCs (Fig. 4). The extent of CD86 upregulation did not differ significantly between NHD- and cHCV–pDCs in response to CpG-A (2-fold), CpG-C (8- to 10-fold), loxoribine (6-fold) and imiquimod (6-fold) (Fig. 4a). CpG-C was the stongest stimulus for CD86 upregulation, being significantly more potent than CpG-A in both subject groups and more effective than loxoribine in cHCV–pDCs. Irrespective of whether the responding pDCs were derived from HCV-infected or uninfected subjects, all four agonists induced a slight (1.5-fold) upregulation of HLA-DR and a 2- to 5-fold increase in cell surface CD83 (data not shown).

BDCA-2 was downregulated from the cell surface by all four agonists, with CpG-C again being the most potent, causing significantly stronger downregulation than CpG-A in both NHD- and cHVC-pDCs (Fig. 4b). There was a trend for BDCA-2 downregulation to be less pronounced on cHCV–pDCs but for individual agonists, the difference in response between NHD- and cHCV–pDC was not significant (Fig. 4b).

### Higher doses of the CpG-A ODN increase IFN-α production by cHCV–pDCs

**Fig. 5:**
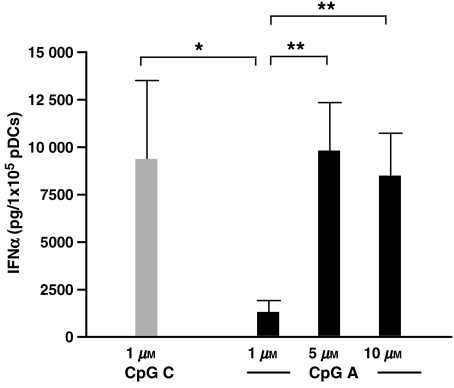
At higher doses the CpG-A ODN stimulates efficient IFN-α production by cHCV-pDCs. Purified pDCs from nine cHCV patients were stimulated with 1 μm CpG-C ODN (grey bar) or 1, 5 or 10 μm CpG-A ODN (solid bars) and IFN-α measured after 20–24 h. Bars represent means ± SEM. IFN-α production in response to 1 μm CpG-A was significantly lower than that induced by 1 μm CpG-C (*P<0.05). Increasing the CpG-A concentration to 5 or 10 μm boosted IFN-α production significantly (5 μm CpG-A > 1 μm CpG-A, **P<0.01; 10 μm CpG-A > 1 μm CpG-A, **P<0.01) reaching levels that did not differ significantly from those induced by 1 μm CpG-C.

In NHD–pDCs, increasing the dose of a class A CpG can augment IFN-α production over a considerably wider concentration range than for CpG-C ODNs (Fig. 1 and [23]). Therefore, in nine cHCV subjects, purified pDCs were stimulated with 1 μm CpG-C and with 1, 5 and 10 μm CpG-A. Again, 1 μm CpG-A failed to induce IFN-α production to the levels achieved by an equimolar amount of the class C CpG ODN (Fig. 5). However, increasing the CpG-A dose to 5 μm induced IFN-α as efficiently as 1 μm CpG-C whilst a further increase to 10 μm CpG-A did not further enhance IFN-α production.

## Discussion

By virtue of their potent secretion of type-1 interferon upon stimulation with synthetic agonists of TLR7 or TLR9, pDCs may be ideal targets for the induction of local, endogenous IFN-α as a novel therapeutic strategy in the treatment of chronic hepatitis C. However, pDCs derived from cHCV patients appear to be profoundly impaired in their ability to produce IFN-α upon *in vitro* stimulation, although these findings are disputed. The present study aimed to address the question of pDC dysfunction in cHCV more definitively, by administering a panel of four agonists of TLR7 or TLR9 to pDCs from patients chronically infected with HCV. The ability of pDCs from cHCV patients to produce IFN-α upon TLR engagement was found to be profoundly but specifically impaired, with phenotypic maturation and the production of other cytokines remaining unperturbed. However, one agonist, a class C CpG ODN, successfully induced robust IFN-α production by cHCV-pDCs, whilst also being a strong stimulus for pDC maturation.

Of the four stimuli tested, both TLR7 agonists (loxoribine and imiquimod) and one TLR9 agonist (the CpG A ODN) were ineffective stimuli for IFN-α production by cHCV-pDCs and yet in pDCs from healthy, uninfected donors, loxoribine and the CpG-A ODN induced robust IFN-α production, although imiquimod remained a weaker stimulus. The failure of three of the four TLR agonists to induce significant IFN-α release from cHCV–pDCs adds to the body of data reporting profoundly depressed IFN-α production by cHCV–pDCs in response to the CpG-A TLR9 agonist ODN2216 [25,27–30], the TLR7/8 agonist, R-848 [30] and to viral stimuli [24,28]. However, other workers have reported normal IFN-α production by cHCV–pDCs [32–35], even when stimulated with the class A CpG, ODN2216.

Given that numbers of circulating pDCs are reduced in cHCV [25,27], some of the conflicting findings regarding pDC dysfunction in cHCV-patients have been attributed to whether IFN-α production has been measured on a per cell basis. However, this explanation is not sufficient. It is particularly pertinent to compare the findings of the current study with those of Decalf *et al.* [35]. Both studies stimulated immunomagnetically purified BDCA4^+^ pDCs with equivalent concentrations of ODN2216. Decalf *et al.* reported normal IFN-α production by pDCs from cHCV patients non-responsive to standard IFN-α2/ribavirin therapy, whereas in the current study cHCV–pDCs failed to respond significantly to CpG-A irrespective of whether they were non-responders or treatment naïve. The opposing findings of these two studies may reflect differences in statistical power; Decalf *et al.* observed highly variable levels of IFN-α production by their cohort of non-responder cHCV patients yet only studied seven patients. Alternatively, the contrasting results from these two studies may be a consequence of the different systems used to measure IFN-α. Decalf *et al.* used Luminex xMAP multiplex technology for which only IFN-α2 specific microspheres are available, whereas the current study used a multi-subtype IFN-α ELISA, capable of detecting 14 of the 15 known human IFN-α subtypes. Since pDCs secrete multiple IFN-α subtypes [12,13,39], with different kinetics of release [40] and differing biological activities [41], measuring only one subtype may not accurately reflect the full capacity of pDCs to produce IFN-α. Indeed, it would be of interest to determine if production of particular IFN-α subtypes is preferentially impaired in cHCV- compared to NHD-pDCs.

The findings of the current study support the concept of depressed IFN-α production by cHCV-pDCs, which occurs in response to a broad spectrum of *in vitro* stimuli. However, for the first time, this study shows that this defect is not universal and can be overcome by certain stimuli, exemplified here by the prototypic class C CpG, M362. It is particularly striking that whilst 1 μm concentrations of the CpG-A and CpG-C ODNs, both agonists of TLR9, induced equivalent levels of IFN-α release from NHD–pDCs, only the CpG-C agonist could induce robust IFN-α production by cHCV–pDCs. However, the suppressed response of cHCV–pDCs to the CpG-A agonist was quantitative rather than qualitative. Thus, when the *in vitro* dose of CpG-A was increased, IFN-α production by cHCV–pDCs reached the levels achieved with a lower dose of CpG-C. This suggests that a threshold of TLR9 signalling must be reached for robust IFN-α production and that due to the action of unknown factors, higher doses of class A ODNs are required to attain this threshold in cHCV–pDCs. In the context of using CpG ODNs as *in vivo* therapeutics, escalating the dose of CpG–A ODNs would be impractical since the molecules aggregate into multimolecular assemblies in a manner which cannot readily be controlled or reproduced [42].

In addition to IFN-α production, engagement of TLR7 and TLR9 on pDCs leads to the release of other cytokines and to immunophenotypic maturation [14,35]. In this study, a multiplex flow cytometric bead assay was used to analyse proinflammatory cytokine production in the same supernatants as used for IFN-α measurement. Although the agonist concentrations and the duration of stimulation were optimized for IFN-α production, TLR ligation also upregulated TNF-α, IL-8, IL-6 and IL-10 production to differing degrees depending on the particular agonist. cHCV–pDCs showed a bias for higher production of TNF-α and IL-8 than their non-diseased counterparts and in no case did levels of cytokine production by cHCV–pDCs fall significantly below those of NHD–pDCs. These findings of normal or slightly elevated production of TNF-α, IL-6 and IL-8 by cHCV–pDCs agree with Decalf *et al.* [35] although deficient IL-6 production by cHCV–pDCs was reported following stimulation with the TLR7/8 agonist R-848 [30]. The extent of immunophenotypic maturation induced by each agonist in this study was comparable for HCV-infected and uninfected pDCs, again with the caveat that agonist concentration and the duration of stimulation were optimized for IFN-α production. This is fully consistent with the maturation response of cHCV–pDCs to influenza virus [35], although in response to the TLR7/8 agonist, R-848, normal upregulation of CD86 expression but impaired upregulation of HLA-DR was reported for cHCV–pDCs [30].

Although a body of data supports the existence of depressed IFN-α production by cHCV–pDCs, the mechanism responsible for this remains to be elucidated. The data presented here point to the defect in cHCV–pDCs being selective, specifically impairing their ability to produce IFN-α whilst sparing proinflammatory cytokine expression and differentiation towards mature APCs. Because proinflammatory cytokine production and phenotypic maturation are unaffected, these data suggest that TLR7/TLR9 expression, affinity and internalization are not abnormal in cHCV–pDCs. Rather, the defect may lie in the ability to signal IFN-α induction. In pDCs, signalling for induction of IFN-α upon TLR7 or TLR9 ligation is mediated by interferon-regulatory factor-7 (IRF-7) [9], whereas expression of pro-inflammatory cytokines and immunophenotypic maturation are controlled by the coordinated actions of nuclear factor κB (NF-κB), MAP kinase [15,16] and IRF-5 [43]. The data presented here are consistent with a selective functional defect in engagement of, or signalling through, the IRF-7 pathway in cHCV–pDCs. The differential response of cHCV–pDCs to CpG-A and CpG-C identified in this study may prove a valuable tool in unravelling the molecular requirements for robust IFN-α induction in cHCV–pDCs.

The data from this *in vitro* study lend support to the emerging concept that in cHCV patients, pDCs are profoundly impaired in their ability to produce IFN-α. However, this need not be an insurmountable hurdle to attempts to target pDCs therapeutically for the production of endogenous IFN-α in HCV patients. As exemplified here by the prototypic class C CpG, M362, with careful selection agonists may be found that can induce robust IFN-α production, proinflammatory cytokine expression and phenotypic maturation in cHCV–pDCs. Agonists identified *in vitro* in this way will require extensive clinical testing to determine if they are capable of boosting pDC function and restoring immune competence in cHCV disease. To date, one class C ODN CpG10101 (Actilon®; Coley Pharmaceutical Group Inc., Wellesley, MA, USA) has undergone clinical testing in cHCV patients refractory to conventional combination therapy. Although Actilon® did not enhance sustained viral clearance in this cohort [44,45], these subjects who had shown resistance to previous exogenous interferon based therapy may have been destined to fail treatment with an immunomodulator that works by inducing endogenous interferons. Critically, the efficacy of type C CpGs in treatment-naïve HCV patients remains untested.

## Authors’ declaration of personal interests

(i) N Libri, S Barker, W Rosenberg and A Semper are employees of iQur Ltd.

(ii) W Rosenberg owns shares in iQur Ltd.

## Abbreviations:

cHCV: chronic HCV
HCV: hepatitis C virus
IFN-α: interferon-alpha
IRF-7: interferon-regulatory factor-7
MFI: mean fluorescence intensity
NF-κB: nuclear factor κB
NHD: normal healthy donors
NR: non-responders
ODN: oligodeoxynucleotide
PBMCs: Peripheral blood mononuclear cells
pDCs: plasmacytoid dendritic cells
TLR7: toll-like receptor 7
